# Crystal structure of tetra­butyl­ammonium bromide–1,2-di­iodo-3,4,5,6-tetra­fluoro­benzene–di­chloro­methane (2/2/1)

**DOI:** 10.1107/S2056989015006593

**Published:** 2015-04-09

**Authors:** Jasmine Viger-Gravel, Ilia Korobkov, David L. Bryce

**Affiliations:** a Department of Chemistry, University of Ottawa, D’Iorio Hall, 10 Marie Curie Private, Ottawa, Ontario, K1N 6N5, Canada

**Keywords:** crystal structure, halogen bonds, short contacts, noncovalent inter­action

## Abstract

The crystallization of a 1:1 molar solution of 1,2-di­iodo-3,4,5,6-tetra­fluoro­benzene (*o*-DITFB) and tetra­butyl­ammonium bromide (*n*-Bu_4_NBr) from di­chloro­methane yielded pure white crystals of a halogen-bonded compound, C_16_H_36_N^+^·Br^−^·C_6_F_4_I_2_·0.5CH_2_Cl_2_ or [(*n*-Bu_4_NBr)(*o*-DITFB)]·0.5CH_2_Cl_2_. The compound may be described as a quaternary system and may be classified as a salt–cocrystal solvate. The asymmetric unit contains one mol­ecule of solvent, two *o*-DITFB mol­ecules, two cations (*n*-Bu_4_N^+^) and two crystallographically distinct bromide ions [θ_I_ **_⋯_**_Br-_ **_⋯_**_I_ = 144.18 (1) and 135.35 (1)°]. The bromide ion is a bidentate halogen-bond acceptor which inter­acts with two covalently bonded iodines (*i.e.* halogen-bond donors), resulting in a one-dimensional polymeric zigzag chain network approximately along the *a* axis. The observed short contacts and angles are characteristic of the non-covalent inter­action [*d*_C—I⋯Br_ = 3.1593 (4)–3.2590 (5) Å; θ_C—I⋯Br_ = 174.89 (7) and 178.16 (7)°]. It is noted that iodine acts as both a halogen-bond donor and a weak CH hydrogen-bond acceptor, while the bromide ions act as acceptors for weak CH hydrogen bonds and halogen bonds.

## Related literature   

The halogen-bonding motif of a polymeric anionic zigzag chain has been described for halogen-bonded compounds of phospho­nium halides and di­iodo­perfluoro­benzenes, see: Abate *et al.* (2009 ▸). For the structure of *o*-DITFB, see: Viger-Gravel (2014 ▸) and of *n*-Bu_4_NBr, see: Elsegood (2011 ▸). The title compound may be classified as a salt–cocrystal solvate, see: Bond (2012 ▸). For weak hydrogen bonds, see: Desiraju & Steiner (1999 ▸).
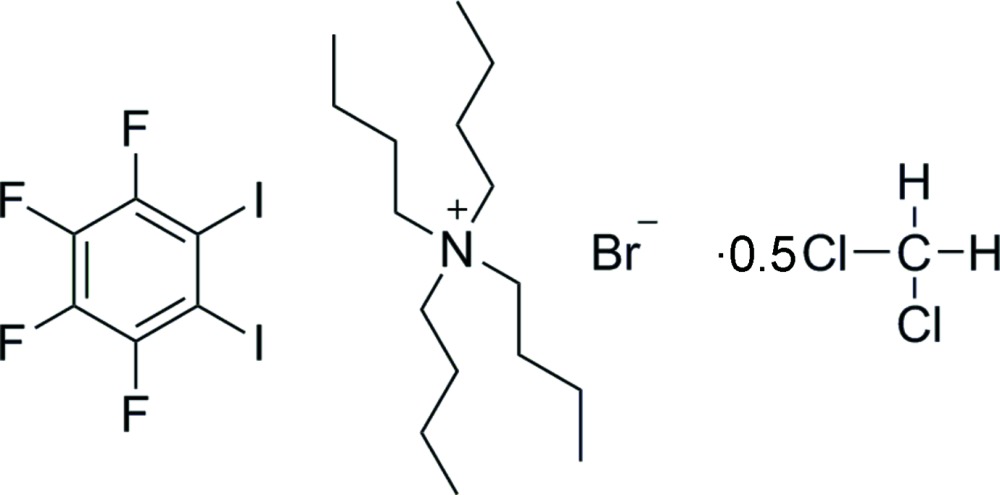


## Experimental   

### Crystal data  

2C_16_H_36_N^+^·2Br^−^·2C_6_F_4_I_2_·CH_2_Cl_2_
*M* *_r_* = 1533.38
Triclinic, 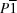

*a* = 13.1654 (3) Å
*b* = 15.0483 (3) Å
*c* = 16.2559 (4) Å
α = 66.668 (1)°
β = 84.654 (1)°
γ = 80.842 (1)°
*V* = 2917.90 (12) Å^3^
*Z* = 2
Mo *K*α radiation
μ = 3.65 mm^−1^
*T* = 200 K
0.24 × 0.24 × 0.12 mm

### Data collection  

Bruker APEXII CCD diffractometer
Absorption correction: multi-scan (*SADABS*; Bruker, 2005 ▸) *T*_min_ = 0.534, *T*_max_ = 0.746
42707 measured reflections
14456 independent reflections
12211 reflections with *I* > 2σ(*I*)
*R*_int_ = 0.022

### Refinement  

*R*[*F* ^2^ > 2σ(*F* ^2^)] = 0.024
*wR*(*F* ^2^) = 0.057
*S* = 1.02
14456 reflections
568 parameters
H-atom parameters constrained
Δρ_max_ = 0.99 e Å^−3^
Δρ_min_ = −0.99 e Å^−3^

### 

Data collection: *APEX2*, Bruker (2005 ▸); cell refinement: *APEX2* and *SAINT* Bruker (2005 ▸); data reduction: *SAINT* and *XPREP* Bruker (2005 ▸); program(s) used to solve structure: *SHELXS03* (Sheldrick, 2008 ▸); program(s) used to refine structure: *SHELXL2013* (Sheldrick, 2015 ▸); molecular graphics: *SHELXTL* (Sheldrick, 2008 ▸); software used to prepare material for publication: *SHELXTL*.

## Supplementary Material

Crystal structure: contains datablock(s) I, New_Global_Publ_Block. DOI: 10.1107/S2056989015006593/gw2150sup1.cif

**Fig. 1.**
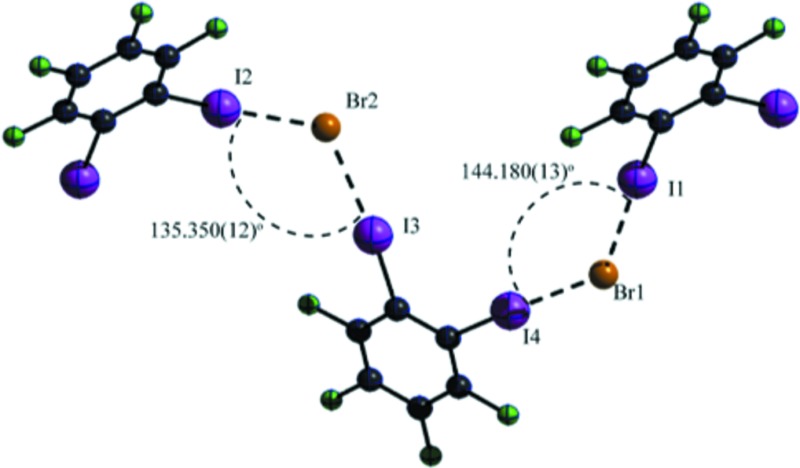
Halogen bonding environment of [(n-Bu4NBr)(o-DITFB)]·CH2Cl2, where iodine is in purple, carbon in black, fluorine in green, and bromine in orange.

**Fig. 2.**
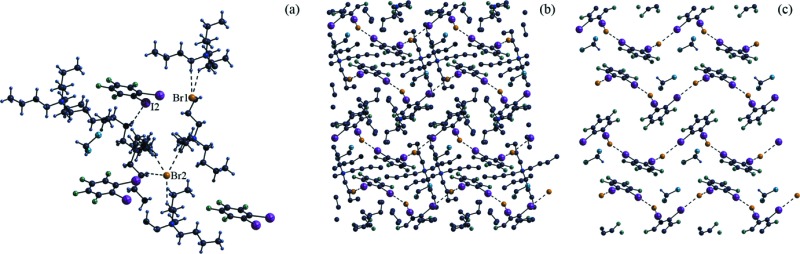
(a) Detail of crystal structure showing selected weak hydrogen bond contacts to bromide and to iodine. (b, c) 2 x 2 x 2 supercell of [(n-Bu4NBr)(o-DITFB)]·CH2Cl2 viewed along the b axis where in (b) the cation is present and in (c) is absent to clarify the image. Rows of the polymeric bromide anionic chains are separated by n-Bu4N+ cations. Hydrogen atoms are not shown for clarity, iodine is in purple, carbon in black, fluorine in green, bromine in orange, and chlorine in blue.

Structure factors: contains datablock(s) I. DOI: 10.1107/S2056989015006593/gw2150Isup2.hkl

n 4 o 2 2 . DOI: 10.1107/S2056989015006593/gw2150fig1.tif
Halogen bonding environment of [(*n*-Bu_4_NBr)(*o*-DITFB)]·CH_2_Cl_2_, where iodine is in purple, carbon in black, fluorine in green, and bromine in orange.

a b c x x n 4 o 2 2 b b c n 4 + . DOI: 10.1107/S2056989015006593/gw2150fig2.tif
(*a*) Detail of crystal structure showing selected weak hydrogen bond contacts to bromide and to iodine. (*b*, *c*) 2 *x* 2 *x* 2 supercell of [(*n*-Bu_4_NBr)(*o*-DITFB)]·CH_2_Cl_2_ viewed along the *b* axis where in (*b*) the cation is present and in (*c*) is absent to clarify the image. Rows of the polymeric bromide anionic chains are separated by *n*-Bu_4_N^+^ cations. Hydrogen atoms are not shown for clarity, iodine is in purple, carbon in black, fluorine in green, bromine in orange, and chlorine in blue.

CCDC reference: 1057419

Additional supporting information:  crystallographic information; 3D view; checkCIF report

## Figures and Tables

**Table 1:** Contacts below the sum of the van der Waals radii involving DITFB (, )

C*X*H	*X*H	C*X*H
C30F2H30*B* ^i^	2.6538(15)	120.189(17)
C33F2H33*A* ^i^	2.8610(19)	170.331(18)
C31F5H31*B* ^i^	2.6222(17)	140.526(17)
C25F7H25*B*	2.518(2)	172.421(16)
C45F7H45*B* ^ii^	2.4890(15)	149.87(3)
C26I2H26*A* ^iii^	3.0204(3)	115.88(7)

Symmetry codes: (i) 1*x*, 1*y*, *z*; (ii) *x*, 1+*y*, *z*; (iii) 1+*x*, *y*, *z*.

**Table 2:** Halogen-bond geometry (, )

C*X* *Y*	*X* *Y*	C*X* *Y*	*Y* *X* *Y*	*Y* *X* *Y*
C1I1Br1	3.2582(5)	177.15(8)	I1Br1I4	144.180(13)
C2I4Br1^iv^	3.1593(4)	178.16(7)		
C1I2Br2^iii^	3.2452(4)	176.83(7)	I3Br2I2	134.350(12)
C2I3Br2^v^	3.2590(5)	174.89(7)		

Symmetry codes: (iv) *x*, 1+*y*, *z*; (iii) 1+*x*, *y*,*z*; (v) *x*, 1+*y*, *z*.

## supplementary crystallographic information

### S1. Experimental

Data collection results for [(*n*-Bu_4_NBr)(*o*-DITFB)]·CH_2_Cl_2_ represent the best data sets obtained in several trials. Crystals were mounted on thin glass fibers using paraffin oil. Prior to data collection, crystals were cooled to 200.15 °K. Data were collected on a Bruker AXS SMART single crystal diffractometer equipped with a sealed Mo tube source (wavelength 0.71073 Å) APEX II CCD detector. Raw data collection and processing were performed with the APEX II software package (BRUKER AXS, 2005). Due to lower symmetry in order to ensure adequate data completeness and redundancy, diffraction data were collected with a sequence of 0.3° ω scans at 0, 90, 180, and 270° in φ. Initial unit cell parameters were determined from 60 data frames with 0.3° ω scan each collected at the different sections of the Ewald sphere. Semi-empirical absorption corrections based on equivalent reflections were applied. In structural models for the compound, hydrogen atom positions were located from the differences in Fourier maps. However, after initial positioning, all hydrogen atoms were constrained to suitable geometries and subsequently treated as idealized contributions during the refinement. All scattering factors are contained in several versions of the SHELXTL program library, with the latest version used being v.6.12.

### S2. Refinement details

Systematic absences in the diffraction data set and unit-cell parameters were consistent with the triclinic P1 (No. 2) space group. Solutions in this centrosymmetric space group yielded chemically reasonable and computationally stable results of refinement. The structure was solved by direct methods, completed with difference Fourier synthesis, and refined with full-matrix least-squares procedures based on *F*^2^. In the structure all molecular fragments are located in general positions.

In the structural model hydrogen atom positions were located from the differences in Fourier maps. However, after initial positioning, all hydrogen atomic positions were constrained to suitable geometries and subsequently treated as idealized contributions. All scattering factors are contained in several versions of the SHELXTL program library, with the latest version used being v.6.12 (Sheldrick, 2008).

### Crystal data

**Table:**

2C_16_H_36_N^+^·2Br^−^·2C_6_F_4_I_2_·CH_2_Cl_2_	*Z* = 2
*M**_r_* = 1533.38	*F*(000) = 1492
Triclinic, *P*1	*D*_x_ = 1.745 Mg m^−^^3^
*a* = 13.1654 (3) Å	Mo *K*α radiation, λ = 0.71073 Å
*b* = 15.0483 (3) Å	Cell parameters from 9968 reflections
*c* = 16.2559 (4) Å	θ = 2.8–28.3°
α = 66.668 (1)°	µ = 3.65 mm^−^^1^
β = 84.654 (1)°	*T* = 200 K
γ = 80.842 (1)°	Block, colourless
*V* = 2917.90 (12) Å^3^	0.24 × 0.24 × 0.12 mm

### Data collection

**Table:**

Bruker APEXII CCD diffractometer	12211 reflections with *I* > 2σ(*I*)
φ and ω scans	*R*_int_ = 0.022
Absorption correction: multi-scan (*SADABS*; Bruker, 2009)	θ_max_ = 28.4°, θ_min_ = 2.0°
*T*_min_ = 0.534, *T*_max_ = 0.746	*h* = −17→17
42707 measured reflections	*k* = −20→20
14456 independent reflections	*l* = −21→21

### Refinement

**Table:**

Refinement on *F*^2^	0 restraints
Least-squares matrix: full	Hydrogen site location: inferred from neighbouring sites
*R*[*F*^2^ > 2σ(*F*^2^)] = 0.024	H-atom parameters constrained
*wR*(*F*^2^) = 0.057	*w* = 1/[σ^2^(*F*_o_^2^) + (0.0216*P*)^2^ + 2.2414*P*] where *P* = (*F*_o_^2^ + 2*F*_c_^2^)/3
*S* = 1.02	(Δ/σ)_max_ = 0.002
14456 reflections	Δρ_max_ = 0.99 e Å^−^^3^
568 parameters	Δρ_min_ = −0.98 e Å^−^^3^

### Special details

**Table:**

Experimental. Data collection is performed with four batch runs at phi = 0.00 ° (600 frames), at phi = 90.00 ° (600 frames), at phi = 180.00 ° (600 frames), and at phi = 270.00 ° (600 frames). A fifth batch run is collected at phi = 0.00 ° (50 frames) to monitor crystal and diffractometer stability. Frame width = 0.30 ° in omega. Data is merged, corrected for decay (if any), and treated with multi-scan absorption corrections (if required). All symmetry-equivalent reflections are merged for centrosymmetric data. Friedel pairs are not merged for noncentrosymmetric data.
Geometry. All e.s.d.'s (except the e.s.d. in the dihedral angle between two l.s. planes) are estimated using the full covariance matrix. The cell e.s.d.'s are taken into account individually in the estimation of e.s.d.'s in distances, angles and torsion angles; correlations between e.s.d.'s in cell parameters are only used when they are defined by crystal symmetry. An approximate (isotropic) treatment of cell e.s.d.'s is used for estimating e.s.d.'s involving l.s. planes.

### Fractional atomic coordinates and isotropic or equivalent isotropic displacement parameters (Å^2^)

**Table:**

	*x*	*y*	*z*	*U*_iso_*/*U*_eq_	
I1	0.24735 (2)	0.72180 (2)	0.14178 (2)	0.04521 (5)	
I2	0.01151 (2)	0.59950 (2)	0.19674 (2)	0.03492 (4)	
I3	0.70784 (2)	−0.18215 (2)	0.37143 (2)	0.03702 (4)	
I4	0.44346 (2)	−0.05201 (2)	0.31053 (2)	0.03407 (4)	
Br1	0.28482 (2)	0.88557 (2)	0.21586 (2)	0.04897 (7)	
Br2	0.81851 (2)	0.63216 (2)	0.32589 (2)	0.03880 (6)	
F1	0.03880 (12)	0.46121 (11)	0.09146 (11)	0.0500 (4)	
F2	0.17634 (14)	0.43539 (12)	−0.03182 (12)	0.0566 (4)	
F3	0.34622 (15)	0.52720 (15)	−0.07434 (13)	0.0693 (5)	
F4	0.37602 (14)	0.64816 (16)	0.00315 (14)	0.0698 (5)	
F5	0.42205 (13)	0.12714 (11)	0.36785 (11)	0.0526 (4)	
F6	0.55057 (16)	0.19756 (12)	0.43805 (12)	0.0635 (5)	
F7	0.74257 (15)	0.10137 (13)	0.48392 (12)	0.0594 (5)	
F8	0.80628 (12)	−0.06286 (13)	0.45697 (12)	0.0572 (4)	
N1	0.91649 (15)	0.31256 (13)	0.48925 (12)	0.0302 (4)	
N2	0.68646 (16)	0.83381 (15)	0.02856 (14)	0.0387 (5)	
C1	0.2196 (2)	0.62142 (19)	0.08822 (17)	0.0392 (5)	
C2	0.13221 (19)	0.57390 (17)	0.11037 (16)	0.0350 (5)	
C3	0.12048 (19)	0.51035 (18)	0.07065 (17)	0.0383 (5)	
C4	0.1907 (2)	0.49549 (19)	0.00793 (18)	0.0429 (6)	
C5	0.2762 (2)	0.5427 (2)	−0.01434 (19)	0.0483 (6)	
C6	0.2901 (2)	0.6044 (2)	0.02608 (19)	0.0468 (6)	
C7	0.54822 (19)	−0.00556 (17)	0.37100 (15)	0.0329 (5)	
C8	0.64711 (19)	−0.05500 (17)	0.39466 (15)	0.0334 (5)	
C9	0.7104 (2)	−0.01755 (19)	0.43258 (18)	0.0408 (6)	
C10	0.6785 (2)	0.06638 (19)	0.44735 (17)	0.0438 (6)	
C11	0.5823 (2)	0.11516 (18)	0.42431 (17)	0.0437 (6)	
C12	0.5173 (2)	0.07841 (17)	0.38746 (16)	0.0385 (5)	
C13	0.94820 (19)	0.21590 (16)	0.56604 (16)	0.0336 (5)	
H13A	1.0118	0.1833	0.5469	0.040*	
H13B	0.8937	0.1738	0.5766	0.040*	
C14	0.9674 (2)	0.22143 (17)	0.65406 (17)	0.0369 (5)	
H14A	0.9033	0.2498	0.6766	0.044*	
H14B	1.0207	0.2644	0.6449	0.044*	
C15	1.0030 (2)	0.11948 (17)	0.72286 (17)	0.0389 (5)	
H15A	0.9492	0.0770	0.7321	0.047*	
H15B	1.0663	0.0910	0.6995	0.047*	
C16	1.0244 (2)	0.1222 (2)	0.81190 (19)	0.0517 (7)	
H16A	1.0463	0.0557	0.8546	0.077*	
H16B	0.9617	0.1500	0.8354	0.077*	
H16C	1.0791	0.1626	0.8033	0.077*	
C17	0.81947 (19)	0.36712 (16)	0.51409 (16)	0.0339 (5)	
H17A	0.8322	0.3769	0.5688	0.041*	
H17B	0.8065	0.4325	0.4653	0.041*	
C18	0.7229 (2)	0.31889 (19)	0.53080 (19)	0.0430 (6)	
H18A	0.7310	0.2568	0.5842	0.052*	
H18B	0.7115	0.3038	0.4786	0.052*	
C19	0.6312 (2)	0.3865 (2)	0.5458 (2)	0.0548 (7)	
H19A	0.6358	0.3885	0.6054	0.066*	
H19B	0.6347	0.4533	0.5002	0.066*	
C20	0.5300 (2)	0.3569 (2)	0.5408 (3)	0.0623 (8)	
H20A	0.4745	0.4035	0.5510	0.093*	
H20B	0.5251	0.2915	0.5867	0.093*	
H20C	0.5240	0.3564	0.4814	0.093*	
C21	0.99919 (19)	0.38009 (17)	0.46600 (16)	0.0351 (5)	
H21A	0.9698	0.4457	0.4245	0.042*	
H21B	1.0157	0.3861	0.5216	0.042*	
C22	1.0985 (2)	0.34942 (19)	0.42390 (18)	0.0403 (6)	
H22A	1.1265	0.2818	0.4621	0.048*	
H22B	1.0851	0.3508	0.3644	0.048*	
C23	1.1766 (2)	0.4183 (2)	0.41343 (18)	0.0458 (6)	
H23A	1.1958	0.4106	0.4736	0.055*	
H23B	1.1446	0.4865	0.3822	0.055*	
C24	1.2730 (3)	0.3988 (3)	0.3611 (3)	0.0737 (10)	
H24A	1.3207	0.4447	0.3559	0.111*	
H24B	1.2546	0.4074	0.3011	0.111*	
H24C	1.3060	0.3318	0.3925	0.111*	
C25	0.89861 (19)	0.28809 (17)	0.41017 (15)	0.0336 (5)	
H25A	0.9643	0.2562	0.3934	0.040*	
H25B	0.8488	0.2402	0.4293	0.040*	
C26	0.8586 (2)	0.37464 (19)	0.32801 (17)	0.0445 (6)	
H26A	0.9047	0.4255	0.3113	0.053*	
H26B	0.7894	0.4028	0.3422	0.053*	
C27	0.8526 (3)	0.3456 (2)	0.24908 (19)	0.0537 (7)	
H27A	0.8369	0.4056	0.1948	0.064*	
H27B	0.9209	0.3127	0.2386	0.064*	
C28	0.7739 (4)	0.2797 (3)	0.2609 (2)	0.0872 (14)	
H28A	0.7751	0.2644	0.2075	0.131*	
H28B	0.7054	0.3123	0.2693	0.131*	
H28C	0.7895	0.2191	0.3136	0.131*	
C29	0.6285 (2)	0.8347 (2)	−0.04838 (18)	0.0444 (6)	
H29A	0.5544	0.8540	−0.0384	0.053*	
H29B	0.6514	0.8860	−0.1043	0.053*	
C30	0.6398 (2)	0.7404 (2)	−0.06305 (18)	0.0450 (6)	
H30A	0.6202	0.6872	−0.0072	0.054*	
H30B	0.7124	0.7228	−0.0790	0.054*	
C31	0.5710 (2)	0.7528 (2)	−0.13814 (19)	0.0513 (7)	
H31A	0.4987	0.7708	−0.1215	0.062*	
H31B	0.5903	0.8072	−0.1932	0.062*	
C32	0.5780 (3)	0.6617 (3)	−0.1580 (2)	0.0667 (9)	
H32A	0.5316	0.6739	−0.2062	0.100*	
H32B	0.5582	0.6076	−0.1040	0.100*	
H32C	0.6489	0.6447	−0.1768	0.100*	
C33	0.80217 (19)	0.81006 (19)	0.01584 (18)	0.0413 (6)	
H33A	0.8166	0.7431	0.0168	0.050*	
H33B	0.8360	0.8098	0.0678	0.050*	
C34	0.8511 (2)	0.8777 (2)	−0.0685 (2)	0.0566 (8)	
H34A	0.8227	0.8742	−0.1213	0.068*	
H34B	0.8340	0.9457	−0.0723	0.068*	
C35	0.9686 (2)	0.8509 (2)	−0.0705 (3)	0.0621 (8)	
H35A	0.9985	0.9031	−0.1214	0.075*	
H35B	0.9956	0.8485	−0.0146	0.075*	
C36	1.0036 (3)	0.7566 (3)	−0.0790 (2)	0.0651 (9)	
H36A	1.0789	0.7439	−0.0787	0.098*	
H36B	0.9800	0.7591	−0.1355	0.098*	
H36C	0.9749	0.7042	−0.0287	0.098*	
C37	0.6580 (2)	0.93582 (19)	0.02917 (18)	0.0426 (6)	
H37A	0.6677	0.9840	−0.0326	0.051*	
H37B	0.5839	0.9442	0.0458	0.051*	
C38	0.7171 (2)	0.9601 (2)	0.0910 (2)	0.0497 (7)	
H38A	0.7891	0.9661	0.0679	0.060*	
H38B	0.7184	0.9069	0.1513	0.060*	
C39	0.6675 (2)	1.0547 (2)	0.0975 (2)	0.0508 (7)	
H39A	0.6566	1.1049	0.0362	0.061*	
H39B	0.5991	1.0451	0.1281	0.061*	
C40	0.7296 (3)	1.0921 (3)	0.1474 (3)	0.0656 (9)	
H40A	0.6921	1.1525	0.1505	0.098*	
H40B	0.7961	1.1053	0.1158	0.098*	
H40C	0.7409	1.0429	0.2082	0.098*	
C41	0.6578 (2)	0.7557 (2)	0.11715 (17)	0.0447 (6)	
H41A	0.6830	0.6911	0.1149	0.054*	
H41B	0.6947	0.7609	0.1651	0.054*	
C42	0.5454 (2)	0.7588 (3)	0.1429 (2)	0.0557 (8)	
H42A	0.5070	0.7522	0.0965	0.067*	
H42B	0.5187	0.8224	0.1469	0.067*	
C43	0.5291 (3)	0.6755 (3)	0.2335 (2)	0.0658 (9)	
H43A	0.5746	0.6788	0.2772	0.079*	
H43B	0.4572	0.6863	0.2549	0.079*	
C44	0.5493 (4)	0.5770 (3)	0.2329 (3)	0.1005 (15)	
H44A	0.5379	0.5288	0.2936	0.151*	
H44B	0.6207	0.5647	0.2129	0.151*	
H44C	0.5026	0.5717	0.1920	0.151*	
C45	0.8462 (3)	0.9249 (3)	0.6717 (3)	0.0774 (11)	
H45A	0.8378	0.9523	0.7185	0.093*	
H45B	0.7938	0.9625	0.6264	0.093*	
Cl1	0.96601 (9)	0.93975 (8)	0.62159 (9)	0.0951 (4)	
Cl2	0.82419 (9)	0.80409 (9)	0.71994 (8)	0.0888 (3)	

### Atomic displacement parameters (Å^2^)

**Table:**

	*U*^11^	*U*^22^	*U*^33^	*U*^12^	*U*^13^	*U*^23^
I1	0.03980 (9)	0.05145 (10)	0.05094 (10)	−0.01695 (7)	0.00179 (7)	−0.02342 (8)
I2	0.03671 (8)	0.03464 (8)	0.03288 (8)	−0.00851 (6)	0.00126 (6)	−0.01166 (6)
I3	0.03450 (8)	0.03702 (8)	0.04262 (9)	−0.00278 (6)	−0.00354 (6)	−0.01896 (7)
I4	0.03396 (8)	0.03321 (7)	0.03537 (8)	−0.00373 (6)	−0.00246 (6)	−0.01365 (6)
Br1	0.04762 (15)	0.04415 (14)	0.05824 (17)	−0.00928 (12)	−0.01439 (13)	−0.01921 (13)
Br2	0.04265 (14)	0.03591 (12)	0.03926 (13)	−0.00107 (10)	−0.00096 (10)	−0.01773 (10)
F1	0.0502 (9)	0.0472 (9)	0.0607 (10)	−0.0161 (7)	0.0022 (8)	−0.0267 (8)
F2	0.0659 (11)	0.0518 (9)	0.0629 (11)	0.0038 (8)	−0.0069 (9)	−0.0369 (9)
F3	0.0623 (12)	0.0857 (14)	0.0656 (12)	−0.0023 (10)	0.0197 (9)	−0.0428 (11)
F4	0.0482 (10)	0.0924 (14)	0.0811 (13)	−0.0296 (10)	0.0273 (9)	−0.0455 (12)
F5	0.0615 (10)	0.0444 (8)	0.0529 (10)	0.0109 (7)	−0.0088 (8)	−0.0251 (7)
F6	0.1002 (15)	0.0406 (9)	0.0601 (11)	−0.0073 (9)	−0.0051 (10)	−0.0307 (8)
F7	0.0777 (12)	0.0600 (10)	0.0569 (10)	−0.0328 (9)	−0.0055 (9)	−0.0305 (9)
F8	0.0427 (9)	0.0709 (11)	0.0721 (12)	−0.0089 (8)	−0.0122 (8)	−0.0397 (10)
N1	0.0384 (11)	0.0249 (8)	0.0298 (10)	−0.0065 (8)	0.0003 (8)	−0.0128 (7)
N2	0.0352 (11)	0.0435 (11)	0.0329 (11)	0.0073 (9)	−0.0077 (8)	−0.0135 (9)
C1	0.0386 (13)	0.0418 (13)	0.0379 (13)	−0.0072 (10)	−0.0002 (10)	−0.0156 (11)
C2	0.0347 (12)	0.0338 (11)	0.0327 (12)	−0.0040 (9)	0.0000 (10)	−0.0094 (10)
C3	0.0371 (13)	0.0334 (12)	0.0420 (14)	−0.0015 (10)	−0.0053 (11)	−0.0126 (10)
C4	0.0492 (15)	0.0374 (13)	0.0424 (14)	0.0044 (11)	−0.0077 (12)	−0.0182 (11)
C5	0.0466 (16)	0.0520 (16)	0.0424 (15)	0.0030 (12)	0.0055 (12)	−0.0194 (13)
C6	0.0385 (14)	0.0522 (16)	0.0479 (16)	−0.0098 (12)	0.0067 (12)	−0.0180 (13)
C7	0.0398 (13)	0.0323 (11)	0.0285 (11)	−0.0077 (10)	0.0013 (9)	−0.0133 (9)
C8	0.0393 (13)	0.0325 (11)	0.0315 (12)	−0.0092 (10)	0.0016 (10)	−0.0148 (9)
C9	0.0413 (14)	0.0446 (14)	0.0391 (13)	−0.0116 (11)	0.0003 (11)	−0.0172 (11)
C10	0.0611 (17)	0.0429 (14)	0.0353 (13)	−0.0238 (13)	0.0012 (12)	−0.0179 (11)
C11	0.0699 (19)	0.0314 (12)	0.0328 (13)	−0.0113 (12)	0.0031 (12)	−0.0151 (10)
C12	0.0497 (15)	0.0326 (12)	0.0309 (12)	−0.0029 (11)	0.0011 (10)	−0.0115 (10)
C13	0.0415 (13)	0.0232 (10)	0.0360 (12)	−0.0028 (9)	−0.0038 (10)	−0.0114 (9)
C14	0.0420 (13)	0.0288 (11)	0.0397 (13)	−0.0023 (10)	−0.0081 (11)	−0.0126 (10)
C15	0.0375 (13)	0.0316 (11)	0.0444 (14)	−0.0035 (10)	−0.0056 (11)	−0.0109 (10)
C16	0.0633 (19)	0.0399 (14)	0.0478 (16)	0.0004 (13)	−0.0224 (14)	−0.0111 (12)
C17	0.0430 (13)	0.0269 (10)	0.0333 (12)	−0.0016 (9)	0.0005 (10)	−0.0146 (9)
C18	0.0451 (15)	0.0359 (12)	0.0485 (15)	−0.0038 (11)	0.0050 (12)	−0.0188 (11)
C19	0.0519 (17)	0.0463 (15)	0.0639 (19)	−0.0016 (13)	0.0139 (14)	−0.0242 (14)
C20	0.0519 (18)	0.0566 (18)	0.076 (2)	0.0023 (14)	0.0054 (16)	−0.0281 (17)
C21	0.0461 (14)	0.0296 (11)	0.0352 (12)	−0.0133 (10)	0.0016 (10)	−0.0161 (10)
C22	0.0419 (14)	0.0409 (13)	0.0433 (14)	−0.0118 (11)	−0.0003 (11)	−0.0194 (11)
C23	0.0490 (16)	0.0524 (15)	0.0399 (14)	−0.0205 (13)	−0.0037 (12)	−0.0166 (12)
C24	0.056 (2)	0.100 (3)	0.074 (2)	−0.034 (2)	0.0123 (17)	−0.038 (2)
C25	0.0423 (13)	0.0328 (11)	0.0325 (12)	−0.0110 (10)	0.0009 (10)	−0.0181 (10)
C26	0.0639 (18)	0.0395 (13)	0.0329 (13)	−0.0169 (12)	−0.0028 (12)	−0.0131 (11)
C27	0.072 (2)	0.0612 (18)	0.0331 (14)	−0.0214 (16)	0.0030 (13)	−0.0207 (13)
C28	0.132 (4)	0.093 (3)	0.053 (2)	−0.062 (3)	−0.013 (2)	−0.026 (2)
C29	0.0418 (14)	0.0528 (15)	0.0347 (13)	0.0069 (12)	−0.0113 (11)	−0.0157 (12)
C30	0.0450 (15)	0.0517 (15)	0.0356 (13)	0.0007 (12)	−0.0046 (11)	−0.0161 (12)
C31	0.0551 (17)	0.0572 (17)	0.0408 (15)	−0.0060 (14)	−0.0102 (13)	−0.0169 (13)
C32	0.075 (2)	0.075 (2)	0.062 (2)	−0.0110 (18)	−0.0065 (17)	−0.0371 (18)
C33	0.0327 (13)	0.0423 (13)	0.0429 (14)	0.0074 (10)	−0.0051 (11)	−0.0140 (11)
C34	0.0508 (17)	0.0497 (16)	0.0546 (18)	0.0076 (13)	0.0063 (14)	−0.0119 (14)
C35	0.0478 (17)	0.0564 (18)	0.076 (2)	−0.0086 (14)	0.0156 (16)	−0.0225 (17)
C36	0.056 (2)	0.068 (2)	0.063 (2)	0.0088 (16)	0.0014 (16)	−0.0234 (17)
C37	0.0373 (13)	0.0464 (14)	0.0414 (14)	0.0091 (11)	−0.0077 (11)	−0.0183 (12)
C38	0.0478 (16)	0.0523 (16)	0.0492 (16)	0.0066 (13)	−0.0120 (13)	−0.0226 (13)
C39	0.0463 (16)	0.0487 (15)	0.0551 (17)	−0.0009 (12)	0.0016 (13)	−0.0206 (13)
C40	0.066 (2)	0.0583 (19)	0.080 (2)	−0.0045 (16)	−0.0077 (18)	−0.0354 (18)
C41	0.0383 (14)	0.0573 (16)	0.0324 (13)	−0.0026 (12)	−0.0042 (10)	−0.0119 (12)
C42	0.0429 (16)	0.074 (2)	0.0429 (16)	−0.0010 (14)	0.0027 (12)	−0.0188 (15)
C43	0.0457 (18)	0.102 (3)	0.0432 (17)	−0.0143 (18)	0.0068 (13)	−0.0208 (18)
C44	0.089 (3)	0.089 (3)	0.095 (3)	−0.019 (2)	0.030 (3)	−0.010 (3)
C45	0.096 (3)	0.073 (2)	0.057 (2)	0.016 (2)	0.0007 (19)	−0.0295 (18)
Cl1	0.0980 (8)	0.0768 (6)	0.1320 (10)	−0.0225 (6)	0.0321 (7)	−0.0670 (7)
Cl2	0.0813 (7)	0.1009 (8)	0.1077 (8)	−0.0284 (6)	0.0117 (6)	−0.0625 (7)

### Geometric parameters (Å, º)

**Table:**

I1—C1	2.109 (3)	C9—C10	1.372 (4)
I2—C2	2.112 (2)	C10—C11	1.362 (4)
I3—C8	2.115 (2)	C11—C12	1.381 (4)
I4—C7	2.112 (2)	C13—C14	1.512 (3)
F1—C3	1.344 (3)	C14—C15	1.528 (3)
F2—C4	1.346 (3)	C15—C16	1.517 (4)
F3—C5	1.342 (3)	C17—C18	1.513 (4)
F4—C6	1.348 (3)	C18—C19	1.517 (4)
F5—C12	1.345 (3)	C19—C20	1.489 (4)
F6—C11	1.341 (3)	C21—C22	1.510 (4)
F7—C10	1.351 (3)	C22—C23	1.526 (3)
F8—C9	1.349 (3)	C23—C24	1.515 (4)
N1—C17	1.517 (3)	C25—C26	1.514 (3)
N1—C13	1.521 (3)	C26—C27	1.522 (4)
N1—C25	1.517 (3)	C27—C28	1.495 (4)
N1—C21	1.527 (3)	C29—C30	1.512 (4)
N2—C33	1.522 (3)	C30—C31	1.522 (4)
N2—C29	1.520 (3)	C31—C32	1.515 (4)
N2—C41	1.516 (3)	C33—C34	1.507 (4)
N2—C37	1.525 (3)	C34—C35	1.537 (4)
C1—C6	1.379 (4)	C35—C36	1.477 (5)
C1—C2	1.395 (3)	C37—C38	1.509 (4)
C2—C3	1.382 (3)	C38—C39	1.508 (4)
C3—C4	1.372 (4)	C39—C40	1.508 (4)
C4—C5	1.371 (4)	C41—C42	1.499 (4)
C5—C6	1.373 (4)	C42—C43	1.534 (4)
C7—C12	1.385 (3)	C43—C44	1.468 (6)
C7—C8	1.398 (3)	C45—Cl2	1.733 (4)
C8—C9	1.383 (3)	C45—Cl1	1.711 (4)
			
C17—N1—C13	111.15 (18)	F8—C9—C8	121.0 (2)
C17—N1—C25	110.59 (18)	C10—C9—C8	121.7 (3)
C13—N1—C25	106.52 (16)	C11—C10—C9	120.0 (2)
C17—N1—C21	106.23 (17)	C11—C10—F7	119.9 (2)
C13—N1—C21	111.40 (18)	C9—C10—F7	120.1 (3)
C25—N1—C21	111.01 (17)	F6—C11—C10	120.5 (2)
C33—N2—C29	111.5 (2)	F6—C11—C12	120.2 (3)
C33—N2—C41	105.38 (18)	C10—C11—C12	119.3 (2)
C29—N2—C41	111.8 (2)	F5—C12—C7	120.7 (2)
C33—N2—C37	111.4 (2)	F5—C12—C11	117.6 (2)
C29—N2—C37	105.28 (18)	C7—C12—C11	121.7 (2)
C41—N2—C37	111.6 (2)	N1—C13—C14	116.31 (18)
C6—C1—C2	118.5 (2)	C13—C14—C15	110.04 (19)
C6—C1—I1	118.13 (19)	C16—C15—C14	111.6 (2)
C2—C1—I1	123.34 (19)	C18—C17—N1	116.13 (18)
C3—C2—C1	118.9 (2)	C17—C18—C19	109.8 (2)
C3—C2—I2	117.59 (18)	C20—C19—C18	113.7 (3)
C1—C2—I2	123.40 (18)	C22—C21—N1	116.31 (18)
F1—C3—C2	121.0 (2)	C23—C22—C21	110.0 (2)
F1—C3—C4	117.2 (2)	C22—C23—C24	112.3 (3)
C2—C3—C4	121.7 (2)	C26—C25—N1	114.90 (19)
F2—C4—C5	119.9 (2)	C25—C26—C27	111.6 (2)
F2—C4—C3	120.8 (3)	C28—C27—C26	114.6 (3)
C5—C4—C3	119.3 (2)	C30—C29—N2	116.9 (2)
F3—C5—C4	119.8 (3)	C29—C30—C31	109.6 (2)
F3—C5—C6	120.7 (3)	C32—C31—C30	113.3 (3)
C4—C5—C6	119.6 (3)	N2—C33—C34	116.3 (2)
F4—C6—C5	117.6 (3)	C35—C34—C33	111.4 (2)
F4—C6—C1	120.5 (3)	C36—C35—C34	114.1 (3)
C5—C6—C1	121.9 (3)	C38—C37—N2	116.5 (2)
C12—C7—C8	118.6 (2)	C37—C38—C39	110.2 (2)
C12—C7—I4	118.16 (18)	C40—C39—C38	113.8 (3)
C8—C7—I4	123.29 (17)	C42—C41—N2	116.1 (2)
C9—C8—C7	118.7 (2)	C41—C42—C43	109.7 (2)
C9—C8—I3	117.76 (19)	C44—C43—C42	115.3 (3)
C7—C8—I3	123.47 (16)	Cl2—C45—Cl1	113.4 (2)
F8—C9—C10	117.4 (2)		
			
C6—C1—C2—C3	1.2 (4)	F6—C11—C12—F5	−1.1 (4)
I1—C1—C2—C3	179.13 (18)	C10—C11—C12—F5	178.2 (2)
C6—C1—C2—I2	−175.3 (2)	F6—C11—C12—C7	178.9 (2)
I1—C1—C2—I2	2.6 (3)	C10—C11—C12—C7	−1.8 (4)
C1—C2—C3—F1	178.4 (2)	C17—N1—C13—C14	−57.7 (3)
I2—C2—C3—F1	−4.8 (3)	C25—N1—C13—C14	−178.2 (2)
C1—C2—C3—C4	−2.3 (4)	C21—N1—C13—C14	60.6 (3)
I2—C2—C3—C4	174.47 (19)	N1—C13—C14—C15	−177.5 (2)
F1—C3—C4—F2	1.2 (4)	C13—C14—C15—C16	179.3 (2)
C2—C3—C4—F2	−178.1 (2)	C13—N1—C17—C18	−64.7 (3)
F1—C3—C4—C5	−178.9 (2)	C25—N1—C17—C18	53.4 (3)
C2—C3—C4—C5	1.8 (4)	C21—N1—C17—C18	173.9 (2)
F2—C4—C5—F3	−1.4 (4)	N1—C17—C18—C19	−174.5 (2)
C3—C4—C5—F3	178.7 (2)	C17—C18—C19—C20	166.0 (3)
F2—C4—C5—C6	179.6 (2)	C17—N1—C21—C22	−170.0 (2)
C3—C4—C5—C6	−0.3 (4)	C13—N1—C21—C22	68.8 (3)
F3—C5—C6—F4	0.0 (4)	C25—N1—C21—C22	−49.7 (3)
C4—C5—C6—F4	179.0 (3)	N1—C21—C22—C23	−174.3 (2)
F3—C5—C6—C1	−179.8 (3)	C21—C22—C23—C24	−172.9 (3)
C4—C5—C6—C1	−0.8 (4)	C17—N1—C25—C26	54.9 (3)
C2—C1—C6—F4	−179.5 (2)	C13—N1—C25—C26	175.8 (2)
I1—C1—C6—F4	2.4 (4)	C21—N1—C25—C26	−62.8 (3)
C2—C1—C6—C5	0.3 (4)	N1—C25—C26—C27	174.5 (2)
I1—C1—C6—C5	−177.8 (2)	C25—C26—C27—C28	67.3 (4)
C12—C7—C8—C9	−0.7 (3)	C33—N2—C29—C30	61.0 (3)
I4—C7—C8—C9	178.92 (18)	C41—N2—C29—C30	−56.7 (3)
C12—C7—C8—I3	−178.46 (17)	C37—N2—C29—C30	−178.1 (2)
I4—C7—C8—I3	1.2 (3)	N2—C29—C30—C31	176.1 (2)
C7—C8—C9—F8	179.7 (2)	C29—C30—C31—C32	179.5 (3)
I3—C8—C9—F8	−2.5 (3)	C29—N2—C33—C34	58.5 (3)
C7—C8—C9—C10	0.0 (4)	C41—N2—C33—C34	−180.0 (2)
I3—C8—C9—C10	177.9 (2)	C37—N2—C33—C34	−58.8 (3)
F8—C9—C10—C11	−179.8 (2)	N2—C33—C34—C35	175.8 (3)
C8—C9—C10—C11	−0.2 (4)	C33—C34—C35—C36	67.8 (4)
F8—C9—C10—F7	0.8 (4)	C33—N2—C37—C38	−50.4 (3)
C8—C9—C10—F7	−179.5 (2)	C29—N2—C37—C38	−171.4 (2)
C9—C10—C11—F6	−179.7 (2)	C41—N2—C37—C38	67.1 (3)
F7—C10—C11—F6	−0.3 (4)	N2—C37—C38—C39	−169.2 (2)
C9—C10—C11—C12	1.0 (4)	C37—C38—C39—C40	−171.8 (3)
F7—C10—C11—C12	−179.6 (2)	C33—N2—C41—C42	−176.2 (3)
C8—C7—C12—F5	−178.4 (2)	C29—N2—C41—C42	−54.9 (3)
I4—C7—C12—F5	2.0 (3)	C37—N2—C41—C42	62.7 (3)
C8—C7—C12—C11	1.6 (4)	N2—C41—C42—C43	180.0 (3)
I4—C7—C12—C11	−178.04 (19)	C41—C42—C43—C44	−69.2 (4)
