# Supplementary figures and images for: Crystal structure of tetra­butyl­ammonium bromide–1,2-di­iodo-3,4,5,6-tetra­fluoro­benzene–di­chloro­methane (2/2/1)
Source: Acta Crystallogr E Crystallogr Commun. 2015 Apr 9;71(Pt 5):o286–7. doi: 10.1107/S2056989015006593 (PMC4420043; doi:10.1107/S2056989015006593)

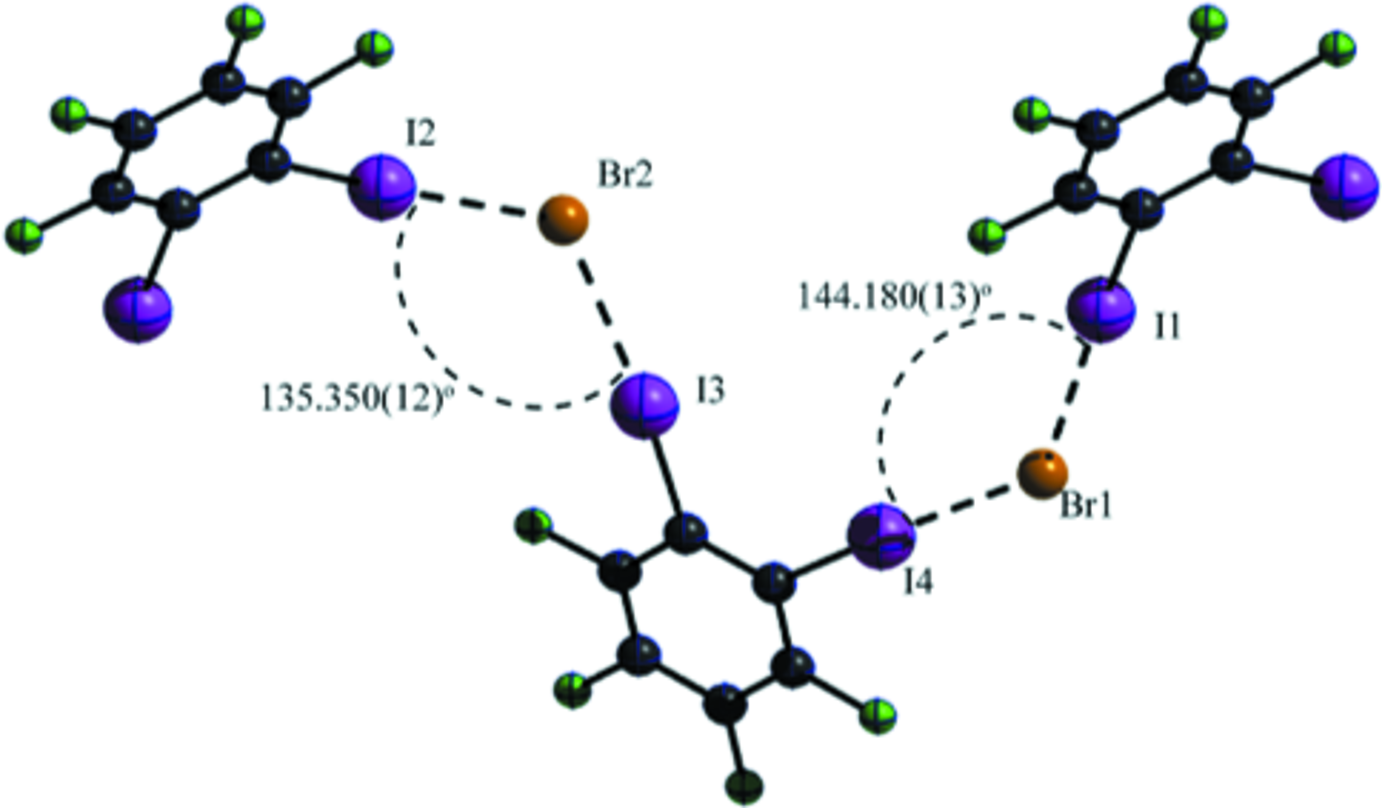

Supplement: Supplementary file 3 [file e-71-0o286-fig1.tif]

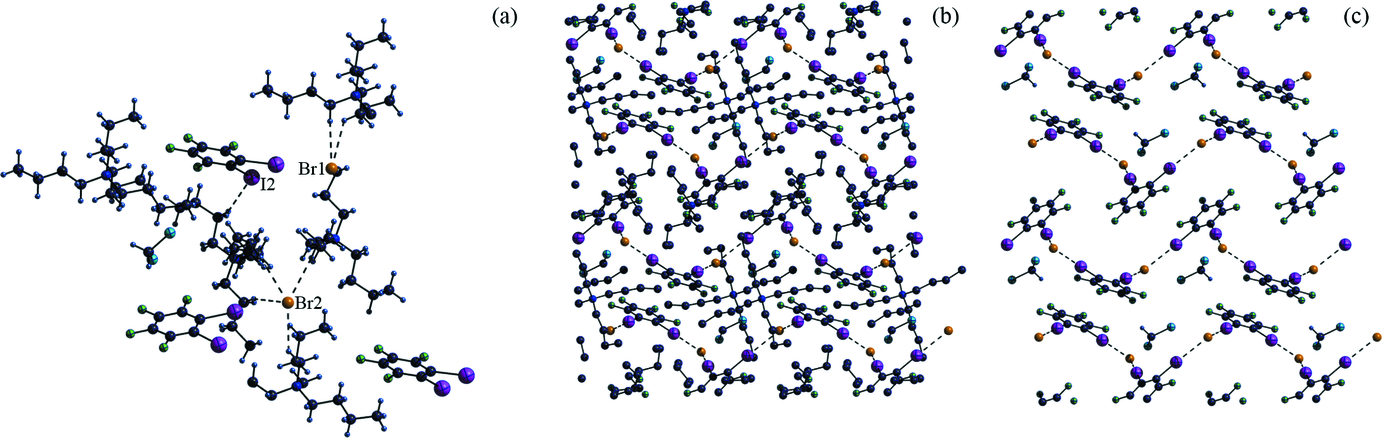

Supplement: Supplementary file 4 [file e-71-0o286-fig2.tif]
